# Transcranial Direct Current Stimulation for Chronic Stroke: Is Neuroimaging the Answer to the Next Leap Forward?

**DOI:** 10.3390/jcm12072601

**Published:** 2023-03-30

**Authors:** Claudia A. Salazar, Wuwei Feng, Leonardo Bonilha, Steven Kautz, Jens H. Jensen, Mark S. George, Nathan C. Rowland

**Affiliations:** 1 Department of Neurosurgery, College of Medicine, Medical University of South Carolina, Charleston, SC 29425, USA; 2 Center for Biomedical Imaging, University of South Carolina, Columbia, SC 29208, USA; 3 Department of Neuroscience, College of Graduate Studies, Medical University of South Carolina, Charleston, SC 29425, USA; 4 Department of Neurology, Duke University School of Medicine, Durham, NC 27710, USA; 5 Department of Neurology, College of Medicine, Emory University, Atlanta, GA 30322, USA; 6 Department of Neurology, College of Medicine, Medical University of South Carolina, Charleston, SC 29425, USA; 7 Department of Health Sciences and Research, College of Health Professions, Medical University of South Carolina, Charleston, SC 29425, USA; 8 Ralph H. Johnson VA Medical Center, Charleston, SC 29401, USA; 9 Department of Radiology and Radiological Science, College of Medicine, Medical University of South Carolina, Charleston, SC 29425, USA; 10 Department of Psychiatry, Medical University of South Carolina, Charleston, SC 29425, USA

**Keywords:** stroke, neuromodulation, transcranial direct current stimulation, magnetic resonance imaging

## Abstract

During rehabilitation, a large proportion of stroke patients either plateau or begin to lose motor skills. By priming the motor system, transcranial direct current stimulation (tDCS) is a promising clinical adjunct that could augment the gains acquired during therapy sessions. However, the extent to which patients show improvements following tDCS is highly variable. This variability may be due to heterogeneity in regions of cortical infarct, descending motor tract injury, and/or connectivity changes, all factors that require neuroimaging for precise quantification and that affect the actual amount and location of current delivery. If the relationship between these factors and tDCS efficacy were clarified, recovery from stroke using tDCS might be become more predictable. This review provides a comprehensive summary and timeline of the development of tDCS for stroke from the viewpoint of neuroimaging. Both animal and human studies that have explored detailed aspects of anatomy, connectivity, and brain activation dynamics relevant to tDCS are discussed. Selected computational works are also included to demonstrate how sophisticated strategies for reducing variable effects of tDCS, including electric field modeling, are moving the field ever closer towards the goal of personalizing tDCS for each individual. Finally, larger and more comprehensive randomized controlled trials involving tDCS for chronic stroke recovery are underway that likely will shed light on how specific tDCS parameters, such as dose, affect stroke outcomes. The success of these collective efforts will determine whether tDCS for chronic stroke gains regulatory approval and becomes clinical practice in the future.

## 1. Introduction

Transcranial direct current stimulation (tDCS) is a noninvasive form of neuromodulation that has shown promise in improving rates of motor recovery following stroke [1,2]. Numerous studies have suggested that the effectiveness of tDCS, particularly as an adjunct to rehabilitation, can even extend to the chronic phase of stroke recovery [1,3,4]. Notwithstanding, a major hurdle of the tDCS field is the high degree of intra- and inter-subject variability in outcomes when tDCS is used for stroke [2,3,4,5,6,7,8,9]. Currently, there remains widespread disagreement regarding the source of variable outcomes resulting from tDCS administration.

Advances in neuroimaging offer potentially fruitful benefits in overcoming this challenge. Individual patient neuroanatomy, connectivity, brain tissue architecture, hemodynamic signals, and neural physiological states can be quantified using commonly available neuroimaging sequences and analytic tools [5,6]. Moreover, many have questioned whether these functional and structural features could account for patient variability in tDCS outcomes and whether such variability relates directly to tDCS-induced motor recovery potential in chronic stroke patients [7].

The idea of using neuroimaging methods, such as fMRI, to elucidate biomarkers corresponding to tDCS response variability has been previously advanced [8]. If individual neuroimaging parameters were known and considered, response to tDCS could become more predictable, thereby reducing variability at scale and leading to broader acceptance (e.g., FDA approval) in the future. This paper reviews preclinical and clinical studies from 2000 to 2022 in which neuroimaging methods were used to investigate mechanisms of tDCS effect on chronic stroke recovery.

PubMed, Google Scholar, ScienceDirect, and Scopus were queried to conduct a comprehensive literature review with the following keywords, individually and in combination: “transcranial direct current stimulation”, “neuroimaging”, “MRI”, “magnetic resonance imaging”, and/or “stroke”. To meet criteria for inclusion in this review, published literature must have met the following requirements:

1. Cortical activation via direct or peripheral stimulation in addition to neuroimaging must have been included in the animal studies.
2. tDCS for chronic stroke survivors (defined as greater than or equal to six months from the time of stroke) in addition to neuroimaging must have been included in the human studies.

Combining tDCS with neuroimaging presents a novel methodological approach for unlocking functional correlates of tDCS mechanisms [10]. The studies outlined in this review (along with others currently ongoing) have the potential to determine the future of tDCS as a clinical treatment for the millions of individuals suffering from chronic stroke.

## 2. Preclinical Studies 

In a 2001 report, Dijkhuizen and colleagues induced unilateral stroke in rodents and examined changes in cortical response to peripheral electrical stimulation (pES) [11]. Functional magnetic resonance imaging (fMRI) cortical activation patterns in a control (sham) group were primarily observed in the hemisphere contralateral to the stimulated forelimb, as expected. In contrast, in the stroke group, three days after infarct, pES-related activation was detected in the contralesional (ipsilateral) hemisphere in various non-motor-related areas (e.g., face, visual, hindlimbs, and barrel field regions of the primary somatosensory cortex). Fourteen days post-stroke, activation was prevalent in both hemispheres, suggesting a dynamic time course of compensatory and reorganizational changes in brain activation following stroke using pES. 

In 2003, the same group investigated cortical hemodynamics and functional recovery using pES in the setting of transient focal ischemia (TFI) [12]. In this study, right middle cerebral artery (MCA) occlusion was performed for two hours in rodents followed by pES of the impaired (contralateral to the lesion) and unimpaired (ipsilateral to the lesion) limbs. The authors calculated a laterality index and demonstrated that, at 24 h and three days post-ischemia, contralesional activity was enhanced when the impaired limb was stimulated, similar to the group’s earlier results. At 14 days post-ischemia, perilesional cortical activity in the ipsilesional hemisphere once again predominated, indicating spatial and temporal dynamics of brain activation. Using neuroimaging, histology, behavioral measures, and neurological deficit scores, the authors were able to establish a direct relationship between cortical insult, degree of tissue injury, and motor recovery. The study concluded that the shift of activation to the contralesional hemisphere elevates as the necrotic burden increases. Although direct stimulation to the brain was not included, these two studies were important in establishing the time course and hemispheric activation changes induced by stroke in rodents.

Yoon et al. (2012) explored the effects of tDCS in relation to immunohistochemical and neuroimaging changes in the rodent brain [12]. Using an MCA TFI model, animals were randomly assigned to one of three stimulation groups: sham, early tDCS (stimulation one day after infarct), or late tDCS (stimulation five days after infarct). The timing of tDCS application was found to have no adverse effects on infarct ratio, volume, or edema index using MRI. However, levels of microtubule-associated protein 2 (MAP-2) and growth-associated protein 43 (GAP-43), both associated with neuroplasticity, differed depending on the timepoint of stimulation. The early tDCS group displayed significant MAP-2 expression, while the late tDCS group exhibited significant GAP-43 enhancement. Both early and late tDCS groups significantly improved in tasks assessing spatial memory, mobility, proprioception, and response to touch. The only group with additional improvement in balance ability was those who received late stimulation. Thus, potential benefits of tDCS on cellular mechanisms and timing of functional recovery have been better defined in this study.

Braun and colleagues (2016) used a neuronal tracer to perform positron emission tomography (PET) imaging in rodents with TFI to investigate how tDCS affects different cell populations within the central nervous system (CNS) [13]. Rodents were randomized to 10 days of tDCS for 15 min at 500μA with either cathodal or anodal polarity. The rodents in the sham group were sedated for 15 min without stimulation. They found that the subventricular zone (SVZ) ipsilateral to the occlusion had higher levels of neuroblast proliferation, suggestive of neurogenesis in response to the infarct. Anodal and cathodal tDCS were employed to address the authors’ secondary question regarding whether tDCS polarity affects different CNS cell types. While tDCS increased neuroblast density radiotracer uptake independent of polarity, oligodendrocytes, which form the myelin sheath, migrated toward the ischemic lesion following cathodal tDCS. In an upper- and lower-extremity motor task, anodal tDCS strengthened gait while cathodal tDCS enhanced both gait and limb strength. In summary, early animal studies revealed distinct activation patterns and cortical hemodynamic changes in response to tDCS. Moreover, these studies helped characterize the myriad cellular processes involved. These investigations established the foundation for using neuroimaging and noninvasive brain stimulation in acute rodent models (see Table 1).

## 3. Early Human Studies Using tDCS for Stroke 

**Figure 1:**
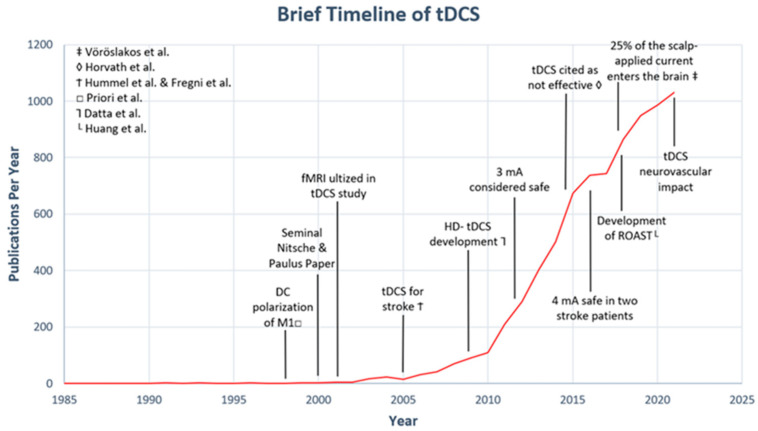
Brief timeline of tDCS. The number of publications per year was calculated in PubMed using the following keyword search: “transcranial direct current stimulation” or “tDCS” [9,16,17,19,20,21,22].

Many questions have been raised about the therapeutic impact and mechanism of action of tDCS since Nitsche and Paulus published their seminal paper describing the technique in 2000 [15]. In that study, Nitsche and Paulus examined the effects of low-intensity current stimulation applied to the scalp and observed that tDCS could induce sustained cortical excitability in the primary motor cortex of healthy controls for up to 90 min [14,15]. Immediately following this publication, many investigations ensued to understand whether tDCS could be used therapeutically for certain clinical populations (see Figure 1 for brief timeline). In 2005, two separate research groups administered tDCS to stroke survivors to address motor impairment [16,17,18]. Hummel et al. were the first to report motor improvement in a single patient with stroke following application of tDCS. In that study, anodal current (1 mA, 20 min) was delivered over the hand knob of the affected primary motor cortex while the cathode was placed supraorbitally on the contralateral scalp. Compared to sham stimulation, the patient demonstrated improvements in motor hand function as measured by the Jebsen–Taylor Hand Function Test (JTT) [18]. The findings reported by Hummel’s team were replicated when Fregni et al. (2005) investigated the effect of cathodal tDCS on the unaffected hemisphere in comparison to anodal tDCS to the affected hemisphere. In that study, six stroke survivors were randomly assigned to one of three groups using a crossover design: anodal tDCS of the affected hemisphere’s motor cortex, cathodal tDCS of the unaffected hemisphere’s motor cortex, or sham treatment. Fregni et al. observed that anodal stimulation of the affected hemisphere and cathodal stimulation of the unaffected hemisphere both significantly improved hand motor function. Fregni’s team extended their findings by demonstrating that cathodal tDCS can produce motor improvements comparable to anodal tDCS [17]. Interestingly, inter-subject variability emerged in the Fregni study. For example, one patient in the anodal tDCS group showed a 3.7% decline in hand function after tDCS, although no explanation was offered as to why this subject responded differently. Other investigators also began noting that tDCS was apparently only effective in some individuals with stroke, prompting many to wonder to what degree tDCS parameters should be optimized for a more consistent response [16,17,18] (see Table 2). 

## 4. The Interhemispheric Inhibition Model Is Introduced Then Challenged

Fregni’s results represent an important development in the field in that they suggest that tDCS might aid stroke recovery by reducing transcallosal inhibition and thus restoring interhemispheric balance in patients recovering from stroke [47]. However, it was not clear whether the rebalancing of hemispheric excitability by tDCS directly facilitated improvements in the upper limbs. Stagg et al. (2012) addressed this question by investigating whether tDCS efficacy is contingent specifically on increased ipsilesional M1 activity [25]. Their study randomly assigned stroke patients to one of three treatment groups: anodal tDCS to the ipsilesional hemisphere; cathodal tDCS to the contralesional hemisphere; or sham. Once each day for three consecutive days, patients were scanned using fMRI while subjected to hand motor tasks. Patients received tDCS for 10 min at 1 mA before and after imaging. Importantly, anodal tDCS patients with larger task-related increases in ipsilesional M1 activation also had larger gains in motor improvement, while the cathodal group did not share this same relationship even though bilateral M1 activation was seen with cathodal tDCS. 

To determine if stroke severity in either hemisphere is a predictor of tDCS effectiveness, Bradnam et al. (2012) adopted a multi-modal approach [23]. In twelve subjects, stroke severity was categorized depending on the extent of white matter tract damage as measured by diffusion tensor imaging (DTI). Subjects with varying upper extremity motor impairments were assessed along with white matter integrity scores based on fractional anisotropy (FA). The authors found that the integrity of white matter tracts is necessary for tDCS to successfully re-establish hemispheric balance. The same year, Lindenberg and colleagues (2012) reported that post-stroke patients’ white matter integrity of the corticospinal tract (CST), also measured by DTI, can predict the recovery of upper extremity motor function in stroke patients [24]. Both works corroborated earlier studies showing that white matter integrity plays a role in facilitating downstream effects of motor recovery, leading to increased use of neuroimaging and tractography in tDCS protocols [24].

Zheng conducted a pilot study in 2015 to further investigate the relationship between integrity of descending motor fibers such as the cortico–rubral–spinal and cortico–tegmental–spinal tracts and post-stroke motor impairment [31,48]. Ten patients received tDCS along with physical and occupational therapy for ten consecutive days. Ten additional patients did not receive either treatment (sham). Zheng found that chronic stroke survivors who participated in the treatment group (tDCS + PT/OT) showed a significant increase in upper extremity Fugl-Meyer Assessment (FMA) scores as opposed to the untreated group. Furthermore, FA values in the treatment group for ipsilesional descending motor fibers increased significantly. Zheng’s results imply that, if properly applied, tDCS could be used to modulate fiber connectivity and FA values.

In 2019, Lee and colleagues used tDCS and repetitive transcranial magnetic stimulation (rTMS) to investigate inter-individual variability among stroke survivors. They enrolled 21 subacute stroke patients who received simultaneous low-frequency rTMS on the contralesional M1 and anodal tDCS on the ipsilesional M1 daily for two weeks. Age-matched healthy controls were also enrolled. fMRI was performed both before and two months after rTMS and tDCS. Laterality indices and lesion maps were analyzed to quantify interhemispheric connectivity. They demonstrated that interhemispheric connectivity was significantly restored after tDCS and rTMS, lending credence to Bradnam’s work [23]. Participants who responded more favorably to tDCS, quantified by higher scores on the FMA-UE, also exhibited a greater interhemispheric balance. 

Notwithstanding, many authors have challenged the role that interhemispheric rebalancing plays in motor recovery from stroke. For example, Stinear and colleagues (2015) used TMS to study corticomotor excitability in subacute stroke survivors and observed that interhemispheric inhibition was stable over time even though ipsilesional activity increased along with motor improvement [49]. The authors speculated that transcallosal inhibition was potentially preserved because the sample contained patients with intact motor cortices. Nevertheless, the concept of establishing an interhemispheric rebalance for motor recovery has been repeatedly questioned. McCambridge et al. (2018) studied modulation of contralesional excitability in ten chronic stroke survivors using tDCS. The authors found that anodal tDCS increased contralesional excitability while cathodal tDCS had no effect. Interestingly, magnetic resonance spectroscopy demonstrated changes in GABA concentration following tDCS, though motor function did not significantly improve with either polarity [50]. Thus, the role of transcallosal effects in motor recovery following stroke as it relates to tDCS remains an open debate [51]. 

## 5. Inter-Subject Variability: Methodological or Biological?

In the studies highlighted in previous sections, paradoxical outcomes in tDCS for stroke were attributed to high inter-subject variability, a phenomenon that has also been noted in tDCS for aphasia [51,52]. Potential factors contributing to response variability have been explored separately in numerous studies: demographics [53]; brain state before, during and after stimulation [54]; pharmacological agents [55], and unique individual neuroanatomy and physiological considerations [56]. To address these criticisms, many experts in the field have concluded that tDCS delivery may require individualized parameters, although how those parameters are computed and what biomarkers, if any, exist to support this approach are still controversial. 

An emerging idea gaining traction is to personalize tDCS dosage using reverse-calculated electric field modeling [57,58,59,60]. The idea stems from the observation that uniform dosing methods, e.g., 2 mA, may underdose some individuals with larger scalp-to-cortex distance or increased cerebrospinal fluid volume, limiting therapeutic response [60,61,62]. These anatomical features may even be exacerbated in stroke given the presence of cortical damage [62], making post-stroke motor rehabilitation an ideal candidate for prospective reverse-calculation dosing for each patient.

The first step in reverse-calculation dosing involves determining an individualized, anatomically accurate tDCS electric field model based on each patient’s T1-weighted (T1w) structural MRI scan [63]. The brain is then segmented, which allows determination of current dispersion based on anatomical characteristics such as skin, cerebrospinal fluid (CSF), skull thickness, and grey and white matter. This processing pipeline helps to create an individualized volumetric finite element model needed for electric field modeling. Using this method, Caulfield and colleagues demonstrated that reverse-calculation modeling can produce the same group average electric field as occurs with uniform 2 mA dosing, while reducing between-individual electric field variance by over 100-fold [26]. 

Caulfield and colleagues subsequently derived a method in which transcranial electrical stimulation (TES) motor threshold (MT) can be used instead of MRI to estimate tDCS dosage based on reverse-calculation modeling [57]. In that study (2020), they utilized transcranial magnetic stimulation (TMS) to determine the MT hotspot. The anodal tDCS electrode was then placed over the MT hotspot, and the cathodal electrode was placed over the deltoid muscle. Based on how much stimulation reached the cortex, the investigators reverse calculated how much to adjust the scalp stimulation dosage to produce the desired electric field at the cortical target [57].

Two key issues remain with personalized dosing. First, the relationship between higher electric field (EF) magnitudes and therapeutic gains is still unclear, as most studies still report maximum dosage delivered instead of dose received. Second, as determined by structural MRI and computer modeling, the conductivity of various head and neck tissues, including skin, bone, and CSF, has a wide functional range. Thus, it is uncertain whether the dosages calculated from computational models will exceed the tolerable limits of current density to the scalp to achieve therapeutically beneficial EFs across the entire stroke population. To address whether tDCS EF normalization could be carried out by instead personalizing the montage used in each subject, Dmochoswki et al. (2013) used high-definition tDCS and MRI to define a target and optimize tDCS electrode configuration. The investigators created a volume conduction model for each patient to determine the electrode montage that would maximize the magnitude of the electric field. Using this approach, they significantly increased the electric field strength by 63%, although a behavioral measurement was not included in that study [26]. Currently, efforts at personalizing tDCS dose are still at an early but promising stage. If computational modeling could help resolve questions about individualizing electric field strength, then this approach might ultimately incorporate neuroimaging as a regular component of tDCS planning and implementation [64]. 

## 6. Randomized Control Trials (RCTs) for tDCS and Stroke Motor Recovery Are Still Evolving

Several Cochrane Reviews investigating the efficacy of tDCS for chronic stroke have been published. In a 2016 report, Elsner et al. analyzed 32 studies involving a total of 748 stroke survivors. The authors found low-to-moderate improvements in activities of daily living in stroke survivors such as holding utensils or picking up a cup following tDCS. In an updated Cochrane Review in 2020, evidence of low-to-moderate efficacy for tDCS therapy was again noted. The authors suggested that future large-scale randomized controlled trials for tDCS were needed to advance the field [24]. Subsequently, in 2021, the Cochrane Review upgraded its findings slightly to conclude that tDCS moderately enhanced outcomes in stroke survivors’ activities of daily living [31]. The updated review indicates the emergence of new evidence regarding use of tDCS in long-term stroke survivors, and in particular, improvements in selective attention [65], visuospatial working memory [65], planning [65], language abilities [66,67,68], faster acquisition of motor skills [68], and gait [69]. Nevertheless, the authors speculated that the inconsistency of tDCS still present in many studies stemmed from the lack of standardization across studies in dosage, voltage, current, stroke volume, and stimulation parameters. The inconsistent parameter settings across studies make it difficult to identify which factors are most associated with the efficacy of tDCS as a therapeutic tool. 

Table 3 lists RCTs with experimental designs that included tDCS for chronic (except for two studies) stroke survivors and neuroimaging. In a randomized, double-blind RCT, Lefebvre and colleagues (2015) recruited nineteen stroke patients and conducted a crossover experimental design: (1) an intervention session where either sham or 1 mA of dual-tDCS (electrodes placed over both M1 regions) were applied during motor learning as measured by the Purdue Pegboard Test; (2) an imaging session one week later in which the subjects were asked to perform the motor skill that was learned in the first session to assess retention ability [70]. The investigators found that dual-tDCS did not improve speed but did significantly enhance the degree of motor skill learning and dexterity of the paretic arm [70]. Sham stimulation resulted in a decrease in Purdue Pegboard Test scores; interestingly, fMRI showed more widespread BOLD activation in the ipsilesional hemisphere after sham than after dual-tDCS [70]. Thus, dual-tDCS-induced fMRI activation is more focal compared to the sham group, suggesting that tDCS shapes the focality of cortical response during a specific task and is important for neuroplastic response distribution in the affected hemisphere. 

In a subsequent study, Darkow and colleagues (2017) recruited sixteen stroke patients with aphasia and asked them to name pictures of common objects during fMRI acquisition. Anodal or sham tDCS was then administered to the ipsilesional M1. They observed that M1 stimulation had no effect on the motor network during a linguistic task, reinforcing the idea that the effects of stimulation are task-dependent and likely driven by task-specific networks rather than a single stimulation site [71]. 

Fortunately, the trend in reporting larger RCT results has continued to increase in recent years. At the time of the writing of this review, according to ClinicalTrials.gov, there are over 280 clinical trials involving tDCS for stroke, 40 of which are currently recruiting specifically for tDCS for motor recovery. Among these is a large Phase II study (ClinicalTrials.gov Identifier: NCT03826030–TRANSPORT 2) being carried out at 12 centers in the US, led by investigators at Duke University. The aim of TRANSPORT 2 is to expand work by Khadka, who reported that tDCS dosing as high as 4 mA is safe and well tolerated by stroke patients in the subacute to chronic phase [78]. TRANSPORT 2 randomizes 129 patients to sham, 2 mA and 4 mA tDCS along with motor assessments, MRI and TMS measurements. With such a large cohort and variety of possible structural defects, it will be important for the study to delineate whether response to escalating tDCS doses is contingent on the integrity of the CST and other important connections, as suggested by earlier studies. Another study, the VERIFY trial (ClinicalTrials.gov Identifier: NCT05338697) will use motor-evoked potential responses to TMS and neuroimaging metrics to validate a predictive tool for upper extremity motor outcomes. 

Although tDCS is generally considered safe, it can result in mild side effects, including burning sensations, tingling, and numbness under the electrodes. While these effects are usually temporary, serious side effects, such as skin irritation, headache, fatigue, and dizziness, can occur in rare cases. Therefore, tDCS should only be administered by trained professionals in a controlled environment, and caution should be exercised in individuals with certain medical conditions or taking specific medications.

## 7. Discussion

As use of tDCS for stroke has evolved, the mechanisms underlying improvement in recovery are still highly debated within the tDCS community. Investigators continue to theorize how optimizing stimulation parameters might render outcomes more consistent. A standardized evaluation protocol is needed for authors and reviewers to assess the impact of features that may influence tDCS response variability. The tDCS community should collaborate to outline key features, including individual differences in brain anatomy, cortical excitability, and stimulation type and duration. A standardized evaluation protocol would enable investigators to compare and interpret the results of tDCS studies more effectively.

Initially, efforts to standardize were focused on the duration of effect, polarity and concerns regarding which stimulated hemisphere produced the strongest effect. As it became clear that establishing hemispheric balance and integrity of white matter tracts were not the only factors important for recovery, attention shifted to understanding the important role that task conditions and functional networks play in determining overall motor effect. Notwithstanding, all of these studies have suffered from some degree of variable effects, and it is now paramount that the tDCS scientific community address how to reduce variable outcomes if the technique is to gain clinical relevance. Increasingly, studies are incorporating neuroimaging in the experimental design in order to better understand these factors. This trend in the use of neuroimaging has the advantage of being complementary to multiple strategies for tDCS delivery, including electric field modeling, dose escalation and many others. 

## 8. New and Unanswered Questions

As mentioned previously, Darkow demonstrated that brain activation following tDCS relies both on the stimulation site and the task performed [71]. Whether targeted brain network activation requires more than just reverse EF modeling or creating montages specific to individual patients remains unknown, but trialing these approaches on a larger scale in the future should be a priority. It is also reasonable to presume that tasks need to be tailored to the network activated. Neuroimaging should be considered essential in all of these experimental design types. Additionally, quantifying the actual tDCS current amplitude delivered to the cortex remains imperative as researchers look for the most precise method to determine the tailored therapeutic dose, as in Caulfield (2020) [58], or the maximum safest dose (2 vs. 4 mA), as in Chhatbar (2017) [79]. Moreover, in the last few years, groups have successfully challenged the conjecture proposed by Nitsche and Paulus in 2001 that tDCS after-effects only last up to 90 min [80]. Lefebvre demonstrated in 2015 that brain activation changes last for up to one week post-dual-tDCS. Those findings were replicated in 2017 [30,72,81]. Thus, the duration and number of administrations of tDCS are also critical considerations, particularly given the portability of the technique which allow it to be delivered both during rehabilitation and at home.

## 9. Future Considerations in Neuroimaging

Stroke can damage descending motor fiber tracts such as the CST [81,82,83], which is a crucial anatomical substrate for voluntary motor function. Efficacy of tDCS is dependent on an intact CST, including as it traverses through the internal capsule, to facilitate functional recovery; therefore, measuring CST structural damage in stroke is important [81,82,83]. In post-stroke patients, the FA of the CST is predictive of upper extremity motor recovery [83]. Higher FA values suggest more myelinated axons and alignment of fibers. As discussed in this review, DTI-based measures of the CST, specifically FA and mean diffusivity (MD), are commonly reported in stroke motor recovery studies [81,82,83]. However, a third measure of interest, mean kurtosis (MK), can also be obtained using diffusion kurtosis imaging (DKI); it quantifies the non-Gaussian quality of water diffusion [29]. DKI is a useful probe of microstructure and is sensitive to detecting changes in permeability [83,84,85]. MK data from acute ischemic stroke survivors revealed abnormalities adjacent to the infarct that were not present in other MR imaging sequences, including DTI, indicating MK’s potential utility in guiding tDCS targeting within stroke populations [85,86]. In the future, studies may consider incorporating advanced imaging metrics such as MK in experimental designs to ascertain if these represent additional biomarkers useful in tailoring tDCS approaches.

## 10. Limitations

This study is not devoid of limitations. One major challenge in understanding the heterogeneity of tDCS studies lies in the significant differences among patient cohorts and stimulation parameters. To address this issue, we recommend that future tDCS investigations prioritize the integration of neuroimaging techniques during concurrent stimulation. This approach can identify differences in neural networks and connectivity across samples, shedding light on tDCS response variability. Moreover, while direct current stimulation studies utilizing imaging techniques in preclinical stroke models are presently more limited in comparison to peripheral electrical stimulation, they are crucial to fully comprehend the underlying mechanisms of action and the potential clinical benefits of this technique. Such studies would allow for the identification of optimal target regions and stimulation protocols, potentially leading to improved stroke recovery outcomes. Hence, the continued investigation of direct current stimulation in preclinical stroke models using imaging techniques is imperative to advance our understanding of this technique and its therapeutic potential. Furthermore, when designing and executing studies involving tDCS, it is imperative to consider the side effects and contraindications associated with this intervention. While this review does not encompass such specifics, they warrant careful attention in all tDCS-related research endeavors.

## Figures and Tables

**Table 1:** Animal studies that incorporated tDCS and MRI.

Authors	Sample	Region(s) of Interest	Experimental Design	Key Finding(s)
Dijkhuizen et al. [11] (2001) **	Unilateral MCA stroke in male Sprague-Dawley rats; N = 6	S1fl, M1	Contrast-enhanced fMRIs were administered 3 days and 14 days post-stroke. 4–5 V for 0.5 ms at 3 Hz for 1 min. Stimulation occurred in the right and then the left forelimb.	Stimulation resulted in a significant increase in neuronal activation-induced rCBV in the S1fl and M1. Contrast-enhanced (CBV-weighted) fMRIs with MINO contrast agent enables high temporal spatial resolution when imaging brain activation patterns. Limb dysfunction is related with loss of neural activation in the ipsilesional sensorimotor cortex.
Dijkhuizen et al. [12] (2003) **	Unilateral MCA stroke in male Sprague-Dawley rats; N = 21	S1fl, M1	Contrast-enhanced fMRIs were administered 1 day, 3 days, and 14 days post-stroke. 5 V for 0.5 ms at 3 Hz for 40 s in the right and then the left forelimb.	The change in activation balance toward the contralesional hemisphere increases with the amount of infarct injury. Recovery is related mostly with preservation of activation in the ipsilesional hemisphere.
Yoon et al. [14] (2012)	MCA territory ischemic rats; N = 30	N/A	3 study groups: sham, early tDCS, and late tDCS.	tDCS was applied in acute stroke rats. tDCS can alter neuronal plasticity surrounding the penumbra without aggravating infarct volume.
Braun et al. [13] (2016)	Left MCA territory ischemic male Wistar rats; N = 28	M1	Rats were assessed for baseline values a day before ischemia. MRI was performed 2 days after ischemia. Rats were trained daily on a motor task. tDCS was administered 3 days after ischemia. tDCS was repeated daily for 5 consecutive days, followed by no stim for 2 days, ending with 5 additional days of stim. There were 3 study groups (ctDCS, atDCS, and sham).	tDCS during acute stroke increases neurogenesis and functional recovery post-stroke.

Abbreviations and Acronyms: S1fl = primary somatosensory forelimb, M1 = primary motor cortex, rCBV = relative cerebral blood volume, MINO = monocrystalline iron oxide nanocolloid. ** did not specifically include tDCS as the technique used in the study design, but electrical stimulation was used.

**Table 2:** Human studies that incorporated tDCS and MRI.

Authors	Sample	Type of Study	Region(s) /Parameter of Interest	Experimental Design	Neuroimaging Modality	Electrode Montage	Key Finding(s)
Bradnam et al. [23] (2012)	At least six weeks post subcortical stroke (N = 12)	Cross-over, double-blind design	M1	Participants attended two experimental sessions in which they completed motor tasks, the NIHSS, FMA, and ASH. One week later, they received either ctDCS or sham tDCS.	sMRI and DWI scans	Contralesional ctDCS was applied over the M1 region with a constant current of 1 mA for 20 min. Anode was placed over the contralateral forehead.	First study to show that ctDCS on the contralesional M1 varies among patients and depends on white matter tract integrity from the ipsilesional hemisphere. ctDCS improved motor function in mildly impaired patients and worsened function for moderately to severely impaired patients.
Lindenberg et al. [24] (2012)	Chronic stroke patients (N = 15)	Comparative study	M1	Participants received bihemispheric tDCS and simultaneous physical/occupational therapy for five consecutive days. Participants underwent DTI at baseline.	DTI	The stimulation consisted of 30 min of 1.5 mA direct current with the anode placed over the ipsilesional and the cathode over the contralesional motor cortex.	DTI measures can be used to predict functional potential for motor recovery.
Stagg et al. [25] (2012)	Chronic ischemic and haemorrhagic stroke (N = 17)	Single-blinded	M1, SMA, PMd	Motor task was administered before, during, and after tDCS.	fMRI	atDCS was placed over the M1 region and the ctDCS was placed over the contralateral supraorbital ridge; 1 mA for 10–20 min.	tDCS-induced brain activation changes using fMRI were reported. atDCS improved response times and increased activation in ipsilesional M1, premotor cortex, and SMA.
Dmochowski et al. [26] (2013)	Chronic stroke (N = 8)	Pilot study	Varied among participants	Participants received MRIs, stimulation, and completed word-naming tasks afterwards.	sMRI, fMRI	hdtDCS was used at 2 mA. Cathodes were placed over the right supraorbital region. Anodes were placed over the target region which varied from patient to patient.	This work individualized hdtDCS montage by utilizing MRI-based modeling of tDCS current flow. Optimizing the electrode montage will result in a 64% increase in EF magnitude at the target. Task performance increased by 38% following optimized montage.
Gillick et al. [27] (2014)	Perinatal ischemic stroke in a 10-year-old	Case report	Bihemispheric M1	MRI was acquired for tDCS montage personalization.	sMRI	ctDCS was placed over C3 and atDCS was placed over C4 at 0.7 mA for 10 min.	Study demonstrated the ability to adapt tDCS mA to specific patient anatomy based on computational modeling analyses
Rosso et al. [28] (2014)	MCA stroke participants with aphasia N = 25	Cross-over, single blind design	Broca’s area (BA)	Participants over three months post-stroke received neuroimaging, followed by a naming task, ending with cathodal stimulation.	Functional, structural, and diffusion MRIs	The electrode center was placed over the ascendant ramus of the lateral sulcus. Reference electrode was placed over the contralateral supraorbital region; 1 mA for 15 min.	tDCS can reduce inhibition of the right BA and reinstate normal interhemispheric inhibition when the left BA is damaged.
Jindal et al. [29] (2015)	Chronic stroke in MCA territory (N = 5)	Joint-imaging and tDCS study	CST	NIRS-EEG/tDCS were placed on the patient’s scalp. Fifteen rounds of tDCS were repeated with 30 s “off” periods in between stim session.	NIRS-EEG/tDCS	ctDCS was placed over the F3 region and atDCS was placed over the Cz region, in accordance to the international 10–20 EEG system. tDCS was repeated 15× with 30 s “off” periods at 0.5A/m^2^.	Variability in CST excitability changes to tDCS are highlighted.
Lefebvre et al. [30] (2015)	Chronic stroke participants (N = 19)	Double-blind, cross-over randomized, sham-controlled experiment	M1	Each subject had two sessions: intervention session during which dual tDCS or sham was applied during motor skill learning with the paretic upper limb; and an imaging session one week later, during which participants performed a task.	fMRI	The anode was positioned over the ipsilesional M1 and the cathode over the contralesional M1; 1 mA for 30 min.	In the dual-transcranial DCS series, the enhanced retention of the motor skill learned one week prior was associated with lesser activation in both hemispheres compared to the sham series, especially in the premotor/motor areas of the ipsilesional hemisphere.
Zheng et al. [31] (2015)	Chronic stroke participants with uni-hemispheric stroke (N = 10)	Pilot study	CST; FA; internal capsule, pons	Participants received 10 days of PT/OT while simultaneously receiving tDCS for 30 min.	DTI	The stimulation consisted of 30 min of 1.5 mA direct current with the anode placed over the ipsilesional motor cortex and the cathode over the contralesional motor cortex.	Chronic stroke survivors who participated in the treatment group (tDCS + PT/OT) showed significant increases in FMA-UE. Furthermore, the treatment group displayed significant increases in FA values for the ipsilesional descending motor fibers.
Chen et al. [32] (2016)	First-time MCA ischemic stroke over three months post-stroke (N = 5)	Proof-of-principle pilot study	Precuneus, M1, premotor cortex	Ten sessions of tDCS combined with PT/OT. PT/OT sessions were 60 min with tDCS for 30 min.	rsfMRI, fMRI	atDCS was placed over the C3 or C4 landmark of the 10-20 EEG system depending on the infarcted hemisphere. ctDCS was placed in the opposite C3 or C4 region. tDCS was applied for 30 min at 1.5 mA.	After treatment, there was a reduction in motor impairment. There was an improvement in the ipsilesional M1 and contralesional premotor cortex’s resting-state connectivity.
Sebastian et al. [33] (2017)	Bilateral MCA ischemic stroke (N = 1)	Double-blind, within-subject crossover trial design	RC, LC, SFG, SFG_PFC, MFG_DLPC, MTG_pole, ITG, FG	There were two conditions: “RC tDCS + behavioral treatment (spelling task)” and “sham tDCS + behavioral treatment”. Each condition consisted of 15 consecutive training sessions, 3–5 per week, two months apart.	sMRI, rs-fMRI were acquired at start of study and two months after completion of study (six-month interval between scans)	tDCS was administered for 20 min at 2 mA. atDCS was placed over the RC and ctDCS was placed over the right deltoid muscle.	Stim and sham treatments resulted in improved spelling. However, there was a trend for greater improvement for the stim treatment. Improvements in spelling coincided with increased connectivity in the cerebro–cerebellar network.
Hordacre et al. [34] (2018)	Chronic stroke (N = 10)	Randomized, cross-over trial	M1	EEG was acquired at the first 3 min of the session, EMG was used throughout session. TMS was used to find hand-knob region in M1, some participants received stim and others sham.	sMRI	atDCS over the lesioned M1 and ctDCS over the contralateral orbit at 1 mA for 20 min.	atDCS did not increase corticospinal excitability measured using resting motor threshold and motor-evoked potentials.
Larcombe et al. [35] (2018)	Stroke survivors with lesion to the primary visual cortex (N = 7)	Pilot study	Visual training	Each participant had a visual performance assessment and an fMRI before and after training. Three participants received anodal tDCS and one had no stimulation.	fMRI	Participants received five 20 min sessions. The stimulation group received 1 mA for 20 min.	No participants showed improvement in visual function, and application of tDCS had no effect on visual performance.
Sánchez-Kuhn et al. [36] (2019) * in Spanish	Cerebellar stroke in 64-year-old man	Case report	Cerebellum	The treatment session comprised of 16 sessions of tDCS with neuroimaging and swallowing therapy for dysphagia for a total of four weeks.	sMRI, dMRI	atDCS was placed over the left M1 and ctDCS was placed over the right trapeze at 1mA for 20 min (16 total sessions).	After the treatment session, there was an increase in white matter fibers and connectivity in the left cerebellar peduncle.
Iyer et al. [37] (2019)	Chronic stroke survivors with a single episode of stroke (N = 20)	Exploratory study with a cross-over design	M1, CBv changes in relation to CME	The first session included clinical measures and TMS measurements before and after anodal tDCS. Participants were block randomized into anodal and sham stimulation for sessions 2 and 3.	Transcranial Doppler (TCD) ultrasound	Anode was placed over the lower limb M1 hotspot on the lesioned hemisphere. Cathode was placed over the supraorbital region; 1 mA for 15 min.	Explored neurovascular changes after tDCS of the lower limb M1 in individuals with stroke. They observed no change in CME or CBv parameters due to anodal tDCS in any of the participants.
Lee et al. [38] (2019)	Subacute stroke survivors (N = 21) & age-matched healthy controls (N = 12)	Randomized study	M1	1 Hz rTMS on the contralesional M1 and anodal tDCS on the ipsilesional M1. Participants were classified into responders and non-responders based on the functional improvement of the affected upper extremity after applying NBS.	fMRI	Anode was placed over the ipsilesional M1 and the cathode was placed over the supraorbital area. 2 mA of current was applied for 20 min.	The imbalanced M1 interhemispheric connectivity between affected and unaffected hemispheres in responders was significantly restored.
Abualait et al. [39] (2020)	Stroke patient exhibiting cortical sensation deficits	Double-blind, sham-controlled, single-case study	M1	The participant underwent sham and stimulation. Following that, the patient completed functional measures. Structural and diffusion tensor imaging data were acquired before and after stimulation.	sMRI, DTI	The patient underwent 20 sessions of sham tDCS followed by 30 sessions of tDCS over both M1 cortices. Each session involved 20 min of 2 mA stimulation.	A positive correlation was observed between improved recovery of fine motor skills and higher FA of the CST as well as increased density of gray matter in specific brain regions. Furthermore, the patient with stroke showed functional improvement and structural changes following tDCS.
Kuo et al. [40] (2020)	First-time, unilateral subcortical ischemic stroke survivors (N = 18)	Randomized sham-controlled crossover study	M1	All participants participated in four experimental sessions on separate days: two real and two sham dual-tDCS sessions, which were combined with either TMS or MEG recordings (i.e., TMS + real tDCS, TMS + sham tDCS, MEG + real tDCS, MEG + sham tDCS).	MEG	The anode was placed over the ipsilesional M1, and the cathode over the contralesional M1. Impedance was kept below 5 kΩ. For true stimulation, a 2 mA current was applied for 20 min.	Stroke survivors had decreased excitability in ipsilesional M1 with excessive transcallosal inhibition from the contralesional to ipsilesional hemisphere at baseline compared with controls.
Richard et al. [41] (2020)	Stroke survivors (N = 54)	Randomized double-blind study	Working memory training and age prediction	Participants were randomized to sham or tDCS stimulation. Participants underwent CCT and MRI before and after the intervention.	MRI	tDCS current was 1 mA for a total of 120 min. Anode was placed over F3 and the cathode was placed over O2.	Utilizing brain morphometry for longitudinal brain age prediction is possible for stroke participants. However, there was no notable correlation between brain age and cognitive training outcomes.
Rezaee et al. [42] (2021)	Male chronic ischemic stroke (N = 12)	Methodological report	Cerebellum	Participants were fitted with an fNIRS-EEG/tDCS cap. Two min of functional connectivity data was collected. Participants then performed a VR task.	fNIRS	atDCS was placed at the contralesional side and ctDCS was placed in the ipsilesional side of the dentate nuclei at 2 mA for 15 min.	Feasibility of fNIRS-EEG joint-imaging of ctDCS was established. However, ctDCS effects on the cerebellum were non-significant.
Lee et al. [43] (2022)	Chronic cerebro–vascular disease (N = 26)	Randomized study	Subcortical areas	Design aimed to observe hemodynamic responses based on tDCS. Participants were asked to sit still and stare at a black screen with a plus sign in the middle.	fNIRS with 66 channels	HD-tDCS device was used. The atDCS and ctDCS were located on C3 and C4 of the 10–20 system at 1 mA.	Cortical activity and synchronization were present each in tDCS trial, followed by a sudden decrease in cortical activity and synchrony.
Kalloch et al. [44] (2022)	Stroke participants (N = 88)	Simulation study	Electric Field	Participants were assigned to four groups of increasing lesion load. They aimed to quantify the change of electrical properties of white matter lesions.	T1 & T2W FLAIR	All simulations were conducted using a bihemispheric electrode. Setup at 2 mA over the 10–20 coordinates C3 & C4 and a frontal–occipital setup over the coordinates FPZ & OZ.	White matter lesions do not perturb the electric field and can be omitted when modeling participants with low to medium lesion load.
Hua et al. [45] (2022)	Participants with poststroke memory impairment (N = 60)	Randomized study	White matter tract FA	Lesion location and memory severity were assessed.	sMRI, DTI	Anodal tDCS of the frontal lobe; parameters were not mentioned.	FA values of the infarct foci and frontal lobe can be used to identify the degree of memory impairment.
Yuan et al. [46] (2022)	Chronic stroke participants with unilateral infarcts (N = 13)	Randomized study	Brain activity in sensori-motor region	Participants completed a motor task in the MRI. After that, rs-MRI analysis was performed before, during, and after. Graph theory analysis of the whole brain was conducted.	tACS-fMRI	Electrodes were placed over the ipsilesional M1 and contralesional supraorbital ridge. One mA was delivered for 20 min by an MRI-compatible DC stimulator.	Functional interaction between the brain regions involved in executive control and SMN regions is facilitated by 20 Hz tACS.

Abbreviations and Acronyms: Neuroimaging Modalities: sMRI = structural MRI, DWI = diffusion weighted imaging, rs-fMRI = resting state functional magnetic resonance imaging, fNIRS = functional near-infrared spectroscopy. Stroke Scales: NIHSS = NIH stroke scale, FMA = Fugle-Meyer assessment, ASH = Ashworth spasticity scales, CCT = computerized cognitive training. tDCS: atDCS = anodal tDCS, ctDCS = cathodal tDCS. hdtDCS = high definition tDCS, EF = electric field. Regions of Interest (ROI): M1 = primary motor cortex, S1 = primary somatosensory cortex, SMA = supplementary motor area, PMd = dorsal premotor area, SMN = sensorimotor network, RC = right cerebellum, LC = left cerebellum, SFG = superior frontal gyrus, SFG_PFC = superior frontal gyrus_prefrontal cortex, MFG_DLPC = middle frontal gyrus/dorsolateral prefrontal cortex, MTG_pole = middle temporal gyrus pole, ITG = inferior temporal gyrus, FG = fusiform gyrus, CME = corticomotor excitability. Parameters: CBv = cerebral blood velocity. * = indicates that the manuscript was written in Spanish.

**Table 3:** Randomized Controlled Trails that incorporated tDCS and MRI.

Authors	Sample	Time from Stroke Onset	Type of Study	Region(s) /Parameter of Interest	Experimental Design	Neuroimaging Technique	Electrode Montage	Key Finding(s)
Lefebvre et al. [30] (2015)	Chronic stroke (N = 19)	Over six months post-stroke	Cross-over, double-blind, randomized design with two sessions	SMA, PMd	The series consisted of two sessions: dual-tDCS or sham during motor skill learning, and an imaging session one week later during motor skill task.	sMRI, fMRI	atDCS was positioned over the ipsilesional M1 and ctDCS was positioned over the contralesional M1 at 1 mA for 30 min.	tDCS enhanced motor learning. Revealed fMRI activation supporting long-term retention of motor skill in stim group.
Darkow et al. [71] (2017)	Chronic stroke (N = 16)	>12 months	Cross-over, sham-tDCS, RCT	Left M1	Naming task and tDCS during MRI.	sMRI, fMRI	atDCS was placed over the left representation of the hand M1. ctDCS was placed over the right supraorbital region. Stim at 1 mA for 20 min and sham.	tDCS modulated neural processing. Stim group displayed decrease in activation in the ACC, left insula, and right lingual gyrus.
Lefebvre et al. [72] (2017)	Chronic hemiparetic stroke (N = 22)	Variable	Randomized, placebo-controlled, double-blind, crossover design	M1, SMA, PMd, SMN, Somato-motor network, salience network	Baseline rs-fMRI, a week later bilateral tDCS and sham, after two weeks from baseline a second tDCS session occurred.	rs-fMRI	Anode over the M1 ipsilesional hemisphere and cathode over M1 in the undamaged hemisphere.	No differences in FC in the ROIs. FC increased in the somatomotor network in the stim group.
Welsby et al. [73] (2018) preprint	First-time ischemic stroke (N = 68)	Over six months post-stroke	Double-blind RCT	Ipsilesional M1	Participants were randomized to sham or stim. MRIs were collected, followed by administration of the FMA, EEG, TMS, ARAT, tDCS, and motor task.	sMRI, fMRI, dMRI	Preprogrammed at-home tDCS for 20 min at 1 mA daily for 2 weeks. atDCS over the ipsilesional M1 and ctDCS over the contralateral supraorbital region.	Results are pending and have not been published yet
Carlson et al. [74] (2018)	Children with perinatal stroke (N = 15)	Variable	Double-blind, sham-controlled, RCT	M1	Ten days of customized, goal-directed therapy was paired with cathodal tDCS over contralesional primary motor cortex. Neuronal metabolites in both M1s were measured before and after intervention using fMRI-guided short-echo 3T MRS.	Proton MRS	Cathode over contralesional M1 at 1 mA for 20 min and sham.	Motor performance improvedin both groups and tDCS was associated with greater goal achievement.
Pruvost-Robieux et al. [75] (2021)	Non-lacunar acute ischemic stroke in the MCA territory (N = 45)	Variable	Proof-of-principle; Single-center, prospective, double-blind, sham-controlled RCT	M1	Participants received imaging and were randomized to ctDCS or sham.	MRI, MRA, DWI	ctDCS electrode was placed in ipsilesional M1, and atDCS was placed in the contralateral supraorbital area. Stimulation current was 1.5 mA for 20 min delivered every hr over 6 h.	ctDCS did not result in a significant reduction of infarct growth volume, although there was an apparent trend towards smaller infarct growth in the stim group.
Kolskår et al. [76] (2021)	Chronic stroke participants (N = 48)	Over six months post-stroke	Prospective double-blind RCT	Feasibility of combining CCT and tDCS on working memory	Participants completed an fMRI at three timepoints. They performed a computerized working memory training program. Each participant completed two weekly tDCS stimulation sessions at the hospital, with a total of six tDCS sessions.	fMRI	Participants were randomized to one of two groups, receiving CCT and either (a) tDCS targeting left dorsolateral prefrontal cortex (1 mA), or (b) sham tDCS, with 40s active stimulation (1 mA) before fading out of the current. Stimulation current was 1 mA.	Results revealed increased performance across all trained tasks, with no additional benefit of tDCS. Brain activation prior to the training was not predictive for training outcome, nor was training gains reflected in altered brain activation.
Räty et al. [77] (2022)	Chronic occipital stroke survivors (N = 16) & healthy controls (N = 12)	Over six months post-stroke	Randomized, sham-controlled RCT	74 cortical ROIs	Participants underwent rsfMRI at baseline, after two weeks of rtACS or sham treatment, and two months of treatment-free follow-up.	rtACS and rsfMRI	Electrodes placed supraorbitally while a ctDCS electrode was on the right forearm. Stimulation frequency alternated between 5 and 15 Hz.	rtACS treatment in the given setting did not affect FC.

Abbreviations and Acronyms: RCT = randomized control trial, ACC = anterior cingulate cortex, FMA = Fugle-Meyer Assessment, CCT = computerized cognitive training, rtACS = repetitive transorbital alternating current stimulation, MRS = magnetic resonance spectroscopy.

## Data Availability

No new data were created or analyzed in this study. Data sharing is not applicable to this article.
